# Word Knowledge in L2 Chinese Lexical Inference: A Moderated Path Analysis of Language Proficiency Level and Heritage Status

**DOI:** 10.3389/fpsyg.2022.869368

**Published:** 2022-06-22

**Authors:** Haomin Zhang, Xing Zhang, Chichi Wang, Jie Sun, Zhenxia Pei

**Affiliations:** ^1^The Psycholinguistics Lab, School of Foreign Languages, East China Normal University, Shanghai, China; ^2^School of English Studies, Shanghai International Studies University, Shanghai, China; ^3^School of English Language, Anhui International Studies University, Hefei, China

**Keywords:** morpheme recognition, morpheme discrimination, heritage language, structural sensitivity, Chinese L2 acquisition

## Abstract

This study explored the effect of word knowledge facets (word-general and word-specific knowledge) on second language (L2) Chinese lexical inference by highlighting the moderating effect of language proficiency level and learners’ heritage status. L2 Chinese learners with a mixture of linguistic (low-intermediate and high-intermediate) and cultural (heritage and non-heritage) backgrounds completed a series of word-knowledge measurements as well as a lexical inferencing task. Through a moderated path model, the study demonstrated that word-general knowledge (morphological awareness) and word-specific knowledge (vocabulary knowledge) contributed to L2 Chinese lexical inference. In addition, the study underlined the moderating effect of heritage status on the correlation between word knowledge and lexical inference. Given the distinct patterns between heritage and non-heritage learners, morphological awareness may define the characteristics of reading profiles in the Chinese heritage learner population.

## Introduction

### Word Knowledge and L2 Lexical Inference

Word learning is a process to establish form, meaning and sound connections to understand definitional knowledge (Nagy and Scott, 2000), during which learners explicitly abstract graphic and phonological representations and map semantic information onto these representations. However, knowing a word involves different categories of information, including form, meaning and use (Nation, 2001). Kieffer and Lesaux (2012) investigated the dimensionality of word knowledge and finalized with the distinction of general-specific knowledge, in which word specific knowledge includes breadth and depth of vocabulary knowledge and word-general knowledge involves metalinguistic knowledge about words and their meanings. Learners with sufficient word knowledge tend to better grasp different vocabulary items and understand structural and semantic relationships in complex compound words in word learning. During this process, inference at the lexical level is essential to vocabulary acquisition and subsequently reading comprehension.

Lexical inference, or deriving the meaning of an unknown word, is conceptualized as “making informed guesses as to the meaning of a word, in light of all available linguistic cues in combination with the learner’s general knowledge of the world, her awareness of context and her relevant linguistic knowledge” (Haastrup, 1991, p. 13). Unlike other types of inference relevant to reading comprehension, learners are supposed to extract word-internal information and then activate contextual information in the same sentence containing the given word. Furthermore, various cognitive decisions are made in the process of inferencing. To date, extant studies have investigated different contributing factors in the inferencing process. First, L2 learners do not always make the guesswork on unfamiliar words, especially if they consider that the word does not hinder their comprehension (Bensoussan and Laufer, 1984; Parry, 1993). A few researchers have also indicated that even when learners make attempts, the ability to achieve a successful inference appears to be different (Haastrup, 1991; Paribakht and Wesche, 1999). Furthermore, although L2 vocabulary learning may take place incidentally in reading (Gass, 1999; Hulstijn et al., 1996; Hulstijn, 2003), it deserves further exploration as to how to achieve vocabulary learning and develop inferencing capacities in reading (Huckin and Coady, 1999; Paribakht and Wesche, 2009). In addition, great variability exists in studies on L2 learners’ ability to understand unfamiliar words, even when the surrounding context is conducive to the inference (Bensoussan and Laufer, 1984; Knight, 1994; Pulido, 2004).

An important factor of L2 inferencing capacity is L2 word knowledge. Numerous studies suggest that there is a positive correlation between word knowledge and L2 lexical inference (e.g., Haastrup, 1991; Wang and Wan, 2011). Wang and Wan (2011) found that word knowledge level was positively correlated with inferencing outcomes. However, they further pointed out that when learners read specific types of passages, there was a threshold at the word-knowledge level. If learners reached a certain level of word knowledge, lexical inference would not be confined; otherwise, word knowledge may impose limitations on inferencing. In addition, specific facets of word knowledge, including vocabulary breadth and depth, were also studied extensively (e.g., Nassaji, 2006; Xun and Sun, 2006; Albrechtsen et al., 2008; Marzban and Hadipour, 2012). For example, Nassaji (2006) investigated the relationship between vocabulary depth, inferencing strategy use (e.g., identifying, evaluating, and monitoring strategies) and word-meaning retrieval from context among English learners. The results indicated that those with stronger depth of vocabulary knowledge used certain strategies more frequently and effectively than their weaker counterparts. Marzban and Hadipour (2012) reported that vocabulary breadth and depth both facilitated successful inferencing, and depth knowledge had a greater influence on lexical inferencing.

In addition, researchers also explored the relationship between word-general knowledge and lexical inference in the L2 context. Park (2004) made a systematic attempt to investigate this relationship among Korean-speaking English language learners. In her findings, a salient correlation was found between morphological awareness and inferencing outcomes. More recently, Zhang and Koda (2012) examined the contributions of morphological awareness and lexical inference to reading comprehension among advanced English learners. They discovered that morphological awareness did not make a direct contribution to reading comprehension, instead it influenced comprehension indirectly. However, in L2 Chinese, Chen (2018) focused on L2 learner-related factors and investigated morphological awareness and lexical inference. He further verified that for proficient learners, morphological awareness was related to inferencing capacity while no significant contribution was found among less-skilled learners. Morphological awareness, as a facet of metalinguistic understanding about words and word meanings, entails word meaning inference. Koda (2000) states that morphological awareness includes the ability to segment morphological structures as well as the ability to understand morphemic meanings. Zhang and Koda (2018b) redefined morphological awareness as a combination of structural awareness and functional awareness. Structural awareness refers to the understanding of structural regularity in morphologically complex words while functional awareness represents the ability to retrieve graphosemantic meanings from complex words. Both facets of morphological awareness may be activated in processing unknown words because structural segmentation and semantic retrieval can facilitate inferencing processes.

To summarize, a number of L2 studies have suggested that word knowledge, including vocabulary knowledge and word processing skills, is essential to inferencing capacity. However, few studies have examined both word-general and word-specific knowledge and the distinction between them is underexplored in the extant literature. A few researchers demonstrated that L2 specific semantic knowledge and general metalinguistic awareness can collectively contribute to L2 inferencing capacities as well as comprehension ability across different languages (Kieffer and Lesaux, 2012; Li and Kirby, 2015; Zhang and Koda, 2018a). However, additional empirical studies are needed to unpack the complexity of word knowledge in L2 Chinese lexical inference.

### Learner Attributes in L2 Lexical Inference and Reading

Individual differences of learner attributes largely contribute to success in adult second language attainment (Dörnyei, 2006). Research on learner attributes has examined psychological variables (e.g., motivation, language attitudes) as well as biographical factors (e.g., generation, years of formal study, bilingual status) (Torres et al., 2019). Given the heterogeneity and complexity of L2 Chinese learners (He, 2008; Zhang, 2016), it is critical to disentangle how linguistic and cultural backgrounds affect L2 Chinese reading acquisition. In the present study, we focus on learners’ linguistic competence (proficiency level) and cultural background (heritage status) and investigate how these two factors impact L2 Chinese reading skills.

### Proficiency Level

Previous research has established that learners with high language proficiency are better at deriving the meanings of new words than the weaker counterparts (e.g., Haastrup, 1991; Morrison, 1996; Fraser, 1999; Bengeleil and Paribakht, 2004; Alavi and Kaivanpanah, 2009). Morrison (1996) claimed that for proficient learners, their vocabulary knowledge accounted for the good performance, while Fraser (1999) attributed the better performance to their advanced processing ability and L2 knowledge. Other studies also verified that language proficiency correlated with the use of contextual clues. Haastrup (1991); Chern (1993), and Haynes (1993) examined lexical inferencing in learners with different proficiency and concluded that high-proficiency students were better at using global clues (i.e., those found beyond the sentence that contains the target word), while low-proficiency ones tended to be confined to local contextual cues (i.e., those found in the same sentence). Furthermore, Haastrup (1991) and Haynes (1993) pinpointed that if L2 learners did not arrive at certain language proficiency, the limited vocabulary knowledge would prevent them from using various linguistic cues to derive the meaning of words. Interestingly, Bensoussan and Laufer (1984) investigated whether proficient students could draw on the context more effectively than less proficient students did in lexical inference. They concluded that proficiency level did not influence inferencing ability, and learners all appeared to employ the same strategy: To ignore the unfamiliar words and make wild guess.

In addition to contextual cues, a number of studies focused on the knowledge sources that learners use in their lexical inference. Soria (2001) examined the use of different sources, and found that advanced learners who might succeed in lexical inference preferred contextual cues; by contrast, learners with low proficiency would resort to interlingual knowledge. Kaivanpanah and Alavi (2008) investigated the contribution of grammatical information to lexical inferencing and found that more proficient learners would extensively use L2 linguistic knowledge sources and would further integrate information from other sources, whereas less proficient learners appeared to emphasize the word-to-word translation in comprehension. Tavakoli and Hayati (2011) explored the knowledge sources that Iranian EFL learners used, and found that low-intermediate level students largely resorted to sentence-level grammatical knowledge, while those at the high-intermediate level seemed to use discourse knowledge to make inference, and that high-intermediate learners achieved successful lexical inference with stronger probabilities. Hamada (2014) found that beginning-level L2 English learners tended to use word-internal morphological cues to derive meanings even the morphological clues were not correct. Interestingly, Chen (2018) found that lexical inference was not directly affected by morphological awareness among low-proficiency L2 Chinese learners, thus arguing that the relation between morphological awareness and L2 Chinese lexical inference varied across proficiency groups.

Overall, learners’ language proficiency can influence learners’ use of contextual information and knowledge sources, and indeed has effect on lexical inference. High proficient learners may have stronger lexical inferencing ability, and therefore made more successful inferencing attempts. Previous studies have also confirmed that if learners do not reach a certain level, their limited word knowledge would hinder their utilizing contextual information to understanding unfamiliar words. More recently, Zhang et al. (2019) further consolidated that specific knowledge and general metalinguistic awareness collectively facilitate inferencing capacities in L2 Chinese. However, it still remains unclear as to whether the contributions of word-specific and word-general knowledge vary in word learning and reading abilities according to different language proficiency levels. In addition, given the disparities in language proficiency, the extent to which learning and instruction can be conducted needs further exploration.

### Heritage Status

In addition to language proficiency level, heritage status is an additional factor of learner attributes that may influence literacy learning. Heritage language (HL) is an immigrant language that a speaker has personal relevance and the desire to (re)connect with Wiley (2005). In the U.S context, Valdés (2000) refers to HL speakers as individuals raised in homes where a language other than English (dominant language) is spoken and who are to some degree bilingual in English and the heritage language. They develop HL literacy mainly in the home environment, and receive English literacy instruction when entering school.

In research on alphabetic languages, oral-based phonological awareness and morphological awareness shape early language and literacy acquisition (e.g., Carlisle, 1995; Nagy et al., 2006; Wolter et al., 2009; Kirby et al., 2012; Parshina et al., 2021). Kremin et al. (2019) compared Spanish-English bilingual and English monolingual children to disentangle how various kinds of knowledge influenced bilingual children’s literacy. They found that there were stronger associations between phonological and orthographic representations in bilingual children than that in monolingual children, and that Spanish-English bilinguals seemed to be heavily reliant on English phonological awareness for learning to read in English. These findings suggest that bilingual children can also benefit from their heritage language with additional learning opportunities, as Spanish and English are alphabetic languages, and both emphasize sound-to-print associations in the learning-to-read process.

Different from literacy development in alphabetic languages, Chinese orthography is phonologically opaque, and its graphemes and pronunciations are not directly mapped, which compounds the difficulty of literacy development. Therefore, it is important to scrutinize how language background can impact literacy development in Chinese as a heritage language (CHL) learners. Ke (1998) examined the home background in Chinese in relation to character recognition and production by comparing collegiate CHL learners and non-CHL learners. He found that there were no significant differences in these two tasks, suggesting that heritage language background did not facilitate Chinese character learning. Similarly, Xiao (2006) conducted two consecutive studies among college-level CHL and non-CHL learners, and investigated the differential associations of literacy skills, i.e., oral language skills, grammar, vocabulary knowledge, character production and reading comprehension. The findings indicated that heritage language background was not the facilitative factor in Chinese vocabulary learning and reading comprehension. In a more recent CHL study, Zhang and Koda (2018a) investigated the associations among vocabulary knowledge, morphological awareness and reading comprehension ability in college-level CHL students. The two constructs both facilitated reading comprehension, and more specifically, morphological awareness strengthened the relationship between vocabulary knowledge and reading. Zhang and Koda (2021) further examined cross-linguistic effects and found that dominant-language morphological awareness was closely correlated with lexical inferencing skills in both dominant language (English) and heritage language (Chinese), and these literacy skills can be transferred across languages. These studies suggest that heritage language background does not necessarily contribute to literacy skills development among HL learners, and more refined theoretical and applied justifications should be provided to the heritage language population.

As of now, a lack of studies has added the covariate of heritage status in understanding L2 Chinese higher-order reading development (e.g., inferencing and comprehension). A few studies have reviewed research on teaching Chinese as a second or a foreign language (Ma et al., 2017; Gong et al., 2018). They argued that despite the growing attention to Chinese language education worldwide, more efforts should be undertaken to delve into HL students’ Chinese language learning, and further to explore the similarities and differences between HL learners and non-HL learners. Therefore, the current study aims to scrutinize the moderation of heritage status in L2 Chinese reading acquisition.

Given the theoretical framing and the review of literature, two research questions are formulated: (1) Do word-knowledge facets (word-general and word-specific knowledge) contribute to L2 Chinese lexical inference? (2) Do L2 language proficiency level and heritage status moderate the relationship between word-knowledge and lexical inference in L2 Chinese?

## Methodology

### Participants

A total of 419 Chinese learners (including 133 low-intermediate students and 386 high-intermediate students; 138 non-heritage students and 281 heritage students) participated in this study and they were from three college-level study-abroad programs in Beijing, Shanghai and Guangzhou. The learners’ age ranged from 18 to 32 with a mean age of 22.13. CHL learners had early exposure to spoken Chinese to varying degrees, however, their literacy skills were constrained because of limited access to print material during their childhood. During data collection, they were all placed into the courses of intermediate level by the placement tests at their institutions or standardized proficiency tests. We recruited intermediate learners in the classrooms after obtaining the consent of the study-abroad program coordinators. They were required to have acquired basic linguistic competence in print Chinese, since we tapped into various dimensions of print knowledge. The study-abroad programs were established to provide students with an enriched environment of language and cultural learning. The participants had received intensive training in language skills and taken culture-related courses to develop linguistic competence in context. Data were collected in a class session and approximately 20 students participated in each session. All tasks were randomized in different sessions to eliminate carry-over effects from prior tasks. The total time allotment was 60 min.

### Instruments

#### Word-General Knowledge

##### Morpheme Recognition

The morpheme recognition task was adopted from Ku and Anderson (2003). To eliminate confusion, some adjustments had been made to ensure that all the vocabulary items were within students’ existing print vocabulary knowledge (lower-level vocabulary in the standardized Chinese Proficiency Test/HSK test). This task aimed to investigate whether learners can understand the semantic relation of a disyllabic word to its subcomponent morpheme. For instance, one disyllable word 可怕 (it literally meant “can” and “afraid” in English, but meant “horrible” in Chinese) and one of its segmental morphemes “可” were both demonstrated to the participants through visual stimuli. They were required to determine whether the meaning of “可怕 (horrible)” was related to the meaning of “可.” The morpheme recognition task involved 20 items and the reliability coefficient (α) for this measure was 0.750.

##### Morpheme Discrimination

The morpheme discrimination task was also modeled after Ku and Anderson (2003). This task was to measure learners’ ability to extract part of word information and distinguish the functional components of morphologically complex words. In this section, three compound words were presented to the participants, for example, 海鱼 “sea fish,” 海边 “seaside,”海报 “poster.” It is obvious that these words share the same morpheme “海,” but the word “海报 (poster)” does not bear the meaning “sea.” The participants were expected to circle the word whose morphemic meaning differs from the other two words. 20 items were involved in the morpheme discrimination task, and the reliability coefficient (α) for this part was 0.770.

#### Word-Specific Knowledge

##### Character Knowledge

The character knowledge task probed into learners’ ability to extract the graphic representations (Chinese characters) of visually presented stimuli. The participants were expected to choose the most appropriate Chinese character combinations. For example, an English stimulus “friendship” was presented at first, and then followed with four options: (A) 友情 (friendship), (B) 朋友 (friend), (C) 客人 (guest), (D) 好客 (hospitality). The participants were supposed to select the correct word with an appropriate combination. There were 30 items in the character knowledge task, and the reliability coefficient (α) was 0.763.

##### Definitional Knowledge

The definitional task aimed to investigate learners’ ability to match semantic meanings with visually presented words. The participants were expected to choose the correct meaning for each word. For instance, a word “职员” was demonstrated to the participants at first, and then they were supposed to select the appropriate explanation from the following items: (A) assistant, (B) account, (C) bank, (D) employee. This task included 30 items with ascending difficulty, and its reliability coefficient (α) was 0.816.

#### Lexical Inferencing Ability

The lexical inferencing task was designed to assess learners’ ability to use word-internal and word-external information when they attempted to understand unknown words. Specifically, this task was to explore the utilization of partial word information and contextual cues in deriving word meanings. All the given words were disyllabic compound words and each word involved two elementary level characters from HSK 1 and 2, which was the lowest bands in HSK. However, the participating intermediate learners were unfamiliar with those compound words because all the words combined were beyond the highest level of the HSK. A pilot testing was conducted among 14 English-speaking intermediate learners before the test. They were required to assess the familiarity of the initially selected words, and 16 compound words were finalized for the present research. In this task, each compound word was placed into a sentence and learners are expected to infer the meaning with the given information, including partial word/morphological information and contextual cues. For example, a sentence “我们坐高铁去北京” (we will go to Beijing by ___) with four choices was demonstrated to the participants: 1. Maglev (morphology–, context+); 2. high-speed train (morphology+, context+, correct); 3. tall building (morphology–, context-); 4. high iron (morphology+, context–). The second option should be selected if learners accurately utilize the word-internal and word-external information in the sentence. Sixteen items were included in this task, and the reliability coefficient (α) for this task was 0.747.

## Results

### Descriptive Statistics and Correlational Analysis

The descriptive analysis (Table 1) showed that all measurements had adequate spread and normality based on standard deviations, skewness and kurtosis. The accuracy rate ranged from 74.2% (definitional knowledge) to 80.7% (morpheme recognition). The correlational patterns among the variables are also presented in Table 1. All measurements had significant correlations with each other. Word-general facets had moderate correlations with lexical inference (*r* = 0.340, *p* < 0.001; *r* = 0.587, *p* < 0.001) and word-specific facets had moderate to strong correlations with lexical inference (*r* = 0.525, *p* < 0.001; *r* = 0.624, *p* < 0.001).

**TABLE 1:** Descriptive statistics and correlations of word-knowledge facets and lexical inference.

Descriptive statistics							Correlation matrix				
Variable	*M*	*SD*	Min	Max	Skewness	Kurtosis	1	2	3	4	5
1. Morpheme recognition (20)	16.14	2.95	2	20	–1.85	5.85	–				
2. Morpheme discrimination (20)	15.73	3.44	3	20	–1.24	1.68	0.490***	–			
3. Character knowledge (30)	23.80	4.37	4	30	–0.99	1.44	0.364***	0.564***	–		
4. Definitional knowledge (30)	22.25	5.17	4	30	–0.73	0.09	0.395***	0.634***	0.777***	–	
5. Lexical inference (16)	12.13	2.74	3	16	–0.87	0.32	0.340***	0.587***	0.525***	0.624***	–

Numbers in parentheses represent the maximum scores of all measurements. Word-general knowledge: morpheme recognition and discrimination; word-specific knowledge: character knowledge and definitional knowledge ***p < 0.001.

### Path Analysis With Moderation

**FIGURE 1:**
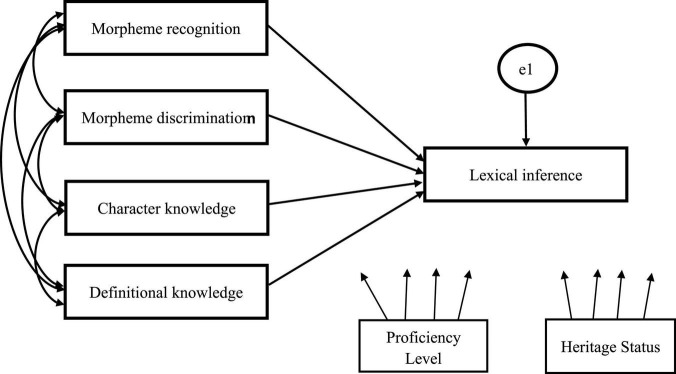
Hypothesized path model.

We first conducted an unmoderated general path analysis to examine the relative contributions of word-knowledge facets to L2 Chinese lexical inference. To further test the hypothesis that the two learner attributes, language proficiency level and heritage status of learners, moderate the relationship between word knowledge facets and lexical inference in L2 Chinese, a moderated path model was proposed to examine the correlations between word knowledge facets and lexical inference among different groups learners. Four grouping variables were entered into the model and the learner subgroups were analyzed based on four different models (c.f. Figure 1). Given that no additional constraints were imposed on each well-defined conceptual model, the overall model was just-identified with saturated model indices (CFI = 1.00, RMSEA = 0.00).

**FIGURE 2:**
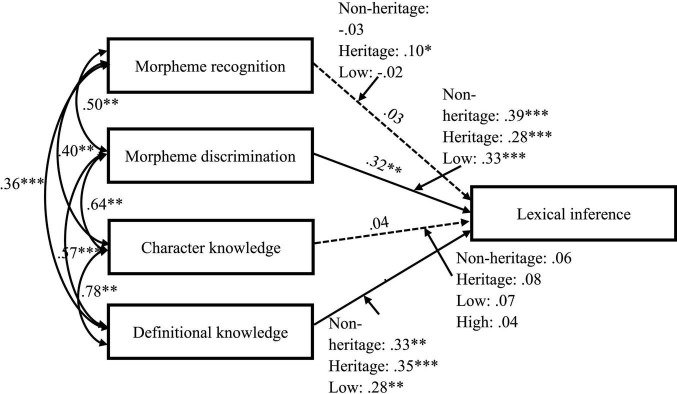
Path diagram of the moderation effect. *p < 0.05, ^**^p < 0.01, ^***^p < 0.001.

Table 2 displays the results of standardized regression weights between the path routes in different groups. Unmoderated correlations and regression weights are directly attached to the path routes in Figure 2. In general, morpheme discrimination and definitional knowledge contributed to lexical inference (β = 0.32, *p* < 0.001; β = 0.38, *p* < 0.001) whereas morpheme recognition and character knowledge have no significant effect on lexical inference. Taking the moderating effects into consideration, the results indicated that the contribution of morpheme recognition to lexical inference was only salient in the heritage group (β = 0.10, *p* < 0.05) and that no groups showed a significant pattern between character knowledge and lexical inference. More specifically, heritage status yielded different relational patterns among the learner groups, however, proficiency level did not generate significant differences of the relation between word knowledge and lexical inference.

**TABLE 2:** Standardized regression weights for measures of moderated path analysis.

Group	Paths			$\hat{\beta }$	*S.E.*	*C.R.(z)*	*p*
Non-heritage	LEXI	< —	MORR	−−0.028	0.054	−−0.379	0.704
	LEXI	< —	MORD	0.391	0.059	4.618	0.000
	LEXI	< —	CHAK	0.062	0.056	0.671	0.502
	LEXI	< —	DEFK	0.331	0.055	3.268	0.001
Heritage	LEXI	< —	MORR	0.095	0.057	1.974	0.048
	LEXI	< —	MORD	0.278	0.052	4.854	0.000
	LEXI	< —	CHAK	0.077	0.047	1.156	0.248
	LEXI	< —	DEFK	0.352	0.040	4.854	0.000
Low	LEXI	< —	MORR	−−0.024	0.078	−−0.272	0.785
	LEXI	< —	MORD	0.328	0.065	3.848	0.000
	LEXI	< —	CHAK	0.070	0.068	0.695	0.487
	LEXI	< —	DEFK	0.282	0.066	2.889	0.004
High	LEXI	< —	MORR	0.060	0.044	1.173	0.241
	LEXI	< —	MORD	0.289	0.051	4.940	0.000
	LEXI	< —	CHAK	0.042	0.042	0.736	0.462
	LEXI	< —	DEFK	0.341	0.042	5.479	0.000

*MORR, Morpheme recognition; MORD, Morpheme discrimination; CHAK, Character knowledge; DEFK, Definitional knowledge; LEXI, Lexical inference.*

## Discussion

The current study generated two interpretable findings. First, word-knowledge facets in general contributed to L2 Chinese lexical inference. More strikingly, the moderator analysis showed that the heritage language learner population benefited relatively more from word-general morphological awareness.

### Vocabulary Knowledge and Morphological Awareness in L2 Chinese Lexical Inference

The findings demonstrated that general cognitive ability (metalinguistic awareness) and specific word knowledge collectively contributed to lexical inference. Vocabulary knowledge has been found to predict L2 lexical inference (Nassaji, 2006). Sufficient text coverage enhances the functionality of inferencing in context because the surrounding context has built surface-level semantic propositions. Additionally, morphological awareness entails the abstraction of morphological structures and morphemic meanings. In line with the prior study (Chen, 2018), the study underscores the utility of morphological awareness in the meaning retrieval of unknown words in L2 Chinese. If we focus on the individual components of word knowledge facets, morpheme discrimination and definitional knowledge yielded significant contributions to L2 Chinese lexical inference. Under a relatively smaller sample size, Zhang et al. (2019) endorsed the positive role of the two components in L2 Chinese inference. Similarly, the current study indicated that morpheme discrimination and definitional knowledge had more salient effects on L2 Chinese inference. Both components highlight the extraction of graphosemantic information. Given the distinctiveness of the Chinese orthography that each character represents one morpheme, graphosemantic understandings of morphemes and words jointly contribute to word learning in context. However, measured character knowledge builds upon the recognition of visual symbols mapped onto meanings and morpheme recognition primarily entails the ability to extract morphemic structures (Zhang et al., 2019). The general insignificance of morpheme recognition and character knowledge indicates that graphic or structural understandings of words may not directly enhance meaning inference.

### Uniqueness of Heritage Learner Population in L2 Chinese Reading

The moderator analysis showed that language proficiency level was not a factor differentiating the correlational patterns while the heritage status presented an interpretable pattern between morphological awareness and lexical inference. First, the non-significant moderation of language proficiency level may be due to the nuanced categorization of the programs. Learners recruited from low-intermediate and high-intermediate classes did not generate a salient difference in their proficiency level, thus justifying the insignificant difference of the correlational strength. Additionally, language proficiency may not affect inferencing strategies (Bensoussan and Laufer, 1984). In the current study, successful lexical inference seemed to rely more upon local semantic cues given that morpheme discrimination and definitional knowledge capitalize on character-level and word-level semantic activation. Furthermore, it is important to highlight that heritage learners benefited more from morphological awareness. Prior comparative studies have found that CHL learners performed differently from non-heritage foreign language learners on language and literacy outcomes (Ke, 1998; Xiao, 2006; Zhang and Koda, 2018b). Home language background provides foundations for early literacy skills including oral language capacity and metalinguistic awareness. Given their expanded oral language repertoire at home and in other situations, HL learners develop their initial morphological recognition ability through oral vocabulary. Constant encounter of high-frequent and morphologically transparent words in oral communication can facilitate their morpheme recognition ability. The current study suggests that both structural recognition and graphosemantic activation enhance lexical inference in the heritage group. Understanding of structural (ir)regularity is also critical to heritage language reading development.

## Conclusion and Implications

The study showed that both word knowledge facets contributed to L2 Chinese lexical inference. Additionally, the findings underscored the moderating effect of heritage status on the correlation between word knowledge and lexical inference. There are a few pedagogical implications for L2 Chinese teaching and learning. First, graphosemantic (character) learning enhances L2 Chinese reading subskill. Given the logographic nature of Chinese, it is highly suggested that students consolidate initial foundational character knowledge and understand graphic representations within multiple-character words. Activities like written character solitaire can be encouraged inside and outside of the classroom, because these gamified activities could stimulate students’ engagement, provide ample opportunities to practice reading skill, and further facilitate their understanding and mastery of Chinese characters (e.g., Huang et al., 2019; Li and O’Rourke, 2022), especially with frequent utilization of character knowledge (Hao, 2018). For example, students can be presented with a written word/character and they need to make words with the antecedent character (今天->天气->气球->球员->员工->工人->人群). Their character knowledge can be further refined and expanded through similar activities.

In addition, the study indicates that heritage students differ from non-heritage students on reading subskill development and that morphological awareness facets provide facilitation to lexical inference among heritage learners. Furthermore, recent studies have also underscored the need to uncover and bridge the differences between HL learners and non-HL learners in word recognition and reading acquisition (e.g., Ma et al., 2017; Gong et al., 2018). Therefore, given their early exposure to spoken language, heritage learners can be explicitly taught to segment bimorphemic and multi-morphemic words through oral-based familiar words. For instance, 机(machine)-derived words (电视机 TV，洗衣机 washer, 相机 camera) can be recognized and retrieved efficiently and HL learners would be able to learn new words based on similar morphological structures. Once the extraction ability develops, reading subskill (lexical inference) can be facilitated.

A few limitations also need further exploration. First, the categorization between intermediate low and intermediate high students did not generate significant patterns. Future studies can include elementary-level and high-level students to further examine the moderating effect of proficiency. Second, lexical inference can be coded as a latent variable with indicators of morphological cues and contextual cues. Graded responses in the lexical inference task can be further coded and multidimensional item analysis can produce the pattern of contextual and morphological factors in predicting lexical inference.

## Data Availability Statement

The raw data supporting the conclusions of this article will be made available by the authors, without undue reservation.

## Ethics Statement

The studies involving human participants were reviewed and approved by the IRB Committee of Carnegie Mellon University. The patients/participants provided their written informed consent to participate in this study.

## Author Contributions

HZ, XZ, CW, JS, and ZP contributed to the conception and design of the study. JS and ZP organized the database. HZ and CW performed the statistical analysis. HZ and XZ wrote the first draft of the manuscript. All authors contributed to the manuscript revision, read, and approved the submitted version.

## Conflict of Interest

The authors declare that the research was conducted in the absence of any commercial or financial relationships that could be construed as a potential conflict of interest.

## Publisher’s Note

All claims expressed in this article are solely those of the authors and do not necessarily represent those of their affiliated organizations, or those of the publisher, the editors and the reviewers. Any product that may be evaluated in this article, or claim that may be made by its manufacturer, is not guaranteed or endorsed by the publisher.
